# miR2Meth: An R package for dissecting DNA methylation heterogeneity at microRNA promoters

**DOI:** 10.1002/qub2.70059

**Published:** 2026-10-19

**Authors:** Ying Shan, Xiaoshan Rong, Andrew E. Teschendorff, Zhen Yang

**Affiliations:** ^1^ Department of Obstetrics and Gynecology Shanghai Pudong Hospital Fudan University Pudong Medical Center Shanghai China; ^2^ Yangzhi Rehabilitation Hospital School of Medicine Tongji University Shanghai China; ^3^ CAS Key Laboratory of Computational Biology Shanghai Institute of Nutrition and Health University of Chinese Academy of Sciences Chinese Academy of Sciences Shanghai China; ^4^ Center for Medical Research and Innovation of Pudong Hospital, and Intelligent Medicine Institute Shanghai Medical College Fudan University Shanghai China

**Keywords:** DNA methylation, heterogeneity, microRNA, multi‐omics, promoter

## Abstract

Aberrant expression of microRNAs (miRNAs) has been found in prevalent cancer types, yet the underlying mechanisms remain largely unexplored due to a lack of analytical tools. Studies have shown that DNA methylation (DNAm) plays a significant role in regulating miRNA expression. Here, we present miR2Meth, an R package to construct DNAm profiles for miRNAs by mapping the probes from the Illumina methylation BeadArray platforms to miRNA promoters. By integrating miRNA expression data, researchers are able to identify aberrant miRNAs associated with promoter methylation dysregulation. This software provides an easy‐to‐use tool to investigate the relationships between miRNAs and DNAm in human diseases. miR2Meth is freely available at GitHub website (github.com/zyangx/miR2Meth).

## 1 INTRODUCTION

MicroRNAs (miRNAs) are a class of small noncoding RNAs, typically 18–22 nucleotides in length, that play a pivotal role in regulating gene expression by targeting specific mRNAs for degradation or translational repression. miRNAs play key roles in various biological processes and are dysregulated in a variety of diseases, including cancer [1]. The research emphasis so far has been on using high‐throughput miRNA expression profiling methods to detect differentially expressed miRNAs between diseased and healthy tissues, which could serve as potential biomarkers. However, the underlying mechanisms of miRNA deregulation have been largely unexplored. Increasing evidence points toward epigenetic modifications in their promoter regions as a key mechanism that controls the highly tissue and cell type‐specific expression pattern of miRNAs [2].

As one of the most important epigenetic marks, DNA methylation (DNAm) is widely known for its role regulating gene expression, for instance, by blocking the recruitment of transcription factors to target genes. The silencing of miRNA expression due to aberrant DNAm events at the promoters has been demonstrated in many cancer types [3]. However, systematic studies of how DNAm influences miRNA transcription have been hampered by a lack of well‐designed analysis tools. Fortunately, the genome‐wide high‐density probe design of recent Illumina Infinium Methylation BeadChip arrays, notably EPIC and EPICv2 assays, cover a large portion of the cis‐regulatory elements in noncoding regions, including those mapping to miRNAs [4].

Here, we introduce miR2Meth, a computational tool to construct DNAm profiles for miRNAs from Infinium BeadChip (Illumina Inc., San Diego, USA) data by mapping their probes to miRNA promoters. This enables researchers to screen biomarkers related to miRNA promoter methylation. By further integrating with miRNA expression analysis, researchers can identify differentially expressed miRNAs that are related to methylation dysregulation. Our tool provides a systematic framework for analyzing aberrant DNAm in miRNAs, thus elucidating their underlying regulatory mechanisms.

## 2 RESULTS

### 2.1 Probe reannotation for constructing DNAm profile of miRNAs

By employing our developed strategy to reannotate Infinium BeadChip probes into human miRNA gene associated promoter regions (5 kb up and downstream of the transcription start site [TSS]: 5 kb ± TSS), we are able to characterize DNAm patterns for miRNA promoters (Figure 1A). For the 450 K assay, a total of 16,272 probe sites were mapped to miRNA gene promoters, and a total of 2056 mature miRNAs from 1285 miRNA genes were assigned at least one probe to their promoter region, with an average of 16.7 probes for each miRNA gene assigned. This covers the majority of the 2588 mature miRNAs that are indexed in miRBase v21 [5]. Whereas, for the EPIC assay, a total of 20,791 probe sites were mapped to the promoter of 2111 mature miRNAs from 1324 miRNA genes, with an average of 20.7 probes assigned to each miRNA gene, and for the EPICv2 assay, a total of 20,906 probe sites were mapped to the promoter of 2107 mature miRNAs from 1318 miRNA genes, with an average of 20.9 probes for each miRNA gene. Users are allowed to define the miRNA promoter region by choosing any length of up to 5 kb up or downstream of the TSS of each miRNA gene, with the region of 2 kb ± TSS recommended. Then, the DNAm level was calculated by using the corresponding values from the cytosine–phosphate–guanine (CpG) sites that fall into the user‐defined miRNA promoter regions. This package provides options to construct methylation matrices for pre‐miRNAs or mature miRNAs. For mature miRNAs, different mature miRNAs (5′‐ and 3′‐) from the same miRNA gene will have the same methylation value.

**FIGURE 1:**
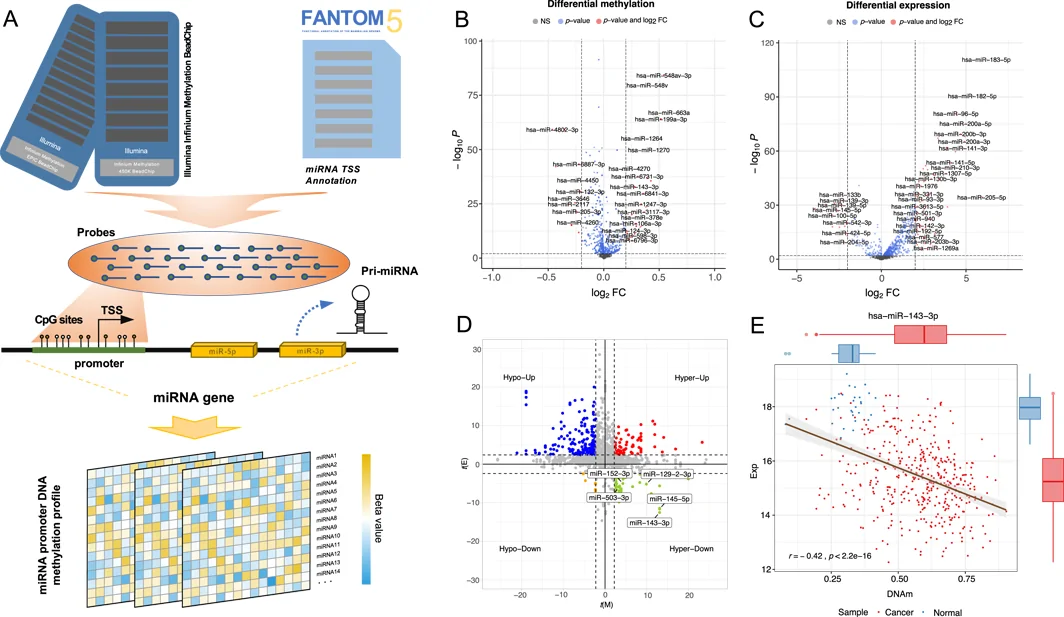
Workflow of miR2Meth and an integrative analysis of differential promoter methylation and differential expression for miRNAs from the TCGA cohort dataset of endometrial cancer. (A) Computational strategy for reannotating Illumina Infinium array probes to construct miRNA promoter methylation profiles. (B) Volcano plot for differential miRNA promoter DNA methylation analysis. (C) Volcano plot for differential expression analysis. (D) Scatter plot of signed *t*‐statistics generated from differential methylation analysis and differential expression analysis. Four groups of miRNAs that present significant differential promoter methylation and expression are labeled by different colors; some miRNAs that have been validated for their associations between promoter methylation and expression by previous studies are indicated. (E) Scatter plot for levels of expression and DNA methylation of hsa‐miR‐143‐3p, *p*‐value is generated from Pearson correlation analysis. CpG, cytosine–phosphate–guanine; DNAm, DNA methylation; Exp, expression; FC, fold change; Hyper‐Down, hypermethylation‐downregulation; Hyper‐UP, hypermethylation‐upregulation; Hypo‐Down, hypomethylation‐downregulation; Hypo‐UP, hypomethylation‐upregulation; NC, not calculated; TCGA, The Cancer Genome Atlas; and TSS, transcription start site.

### 2.2 Software application for case study

The basic workflow of miR2Meth mainly consists of three steps: (1) Data upload and miRNA promoter DNAm profiling construction: This package accepts methylation data stored in a CpG (rows) by sample (columns) matrix with beta values as input; the DNAm matrix with miRNAs (rows) by sample (columns) was constructed by mapping the probes to miRNA promoters. (2) Differential miRNA promoter methylation analysis: The differentially methylated miRNAs can be determined with a phenotype of interest, such as “cancer versus normal,” by using the widely used limma method or methods of *t*‐test or Wilcoxon‐rank sum test, with Benjamini–Hochberg corrected *p*‐values provided. (3) Integrative study with miRNA expression profiling data: with the miRNA expression matrix as input, the differentially expressed miRNAs can be determined with same methods, and then the intersection between the differentially methylated and expressed miRNAs can be selected to evaluate the impact of DNAm of miRNA promoters on their expression.

To determine the validity of the probe reannotation and methylation construction strategy, we applied this tool to a cohort set of endometrial cancer (UCEC) 450 K DNAm data from The Cancer Genome Atlas (TCGA), representing samples of normal and tumor physiology. We used the Beta MIxture Quantile dilation method‐corrected DNAm matrix as input [6]. Here, we only focus on those probes proximal to each miRNA TSS to determine the DNAm status of their promoters. Probes located in the regions with 2 kb upstream and 500 bp downstream from the TSS were retained as miRNA promoter associated. The DNAm profile was constructed by averaging the *β*‐values of the probes mapping to the promoter of each miRNA. This strategy is analogous to that from protein coding genes and has been confirmed by our previous study. Therefore, we constructed promoter methylation profile for 1977 miRNAs in 471 normal and corresponding tumor samples.

We next searched for the miRNAs with differential methylation between normal and tumor tissues. For the miRNA promoter methylation profile constructed, the DNAm levels of 1132 miRNAs showed significant differences between normal and tumor samples at false discovery rate (FDR) < 0.05 by using the limma method (Figure 1B). For comparison, we also screened differentially methylated miRNAs by using *t*‐test and Wilcoxon‐rank sum test methods, with 1359 and 1280 miRNAs obtained, respectively. A significant part of the miRNAs presents common conclusions from the three methods (Supporting Information S1: Figure S1A). To further check the influence of promoter methylation status on miRNA expression, we obtained UCEC miRNA‐seq profiling data which were quantified as reads per million from TCGA. Differential expression analysis was performed between the 33 normal and 538 tumor samples. A total of 751 differentially expressed miRNAs were detected with limma (Figure 1C), and 1479 and 841 miRNAs were identified by using *t*‐test and Wilcoxon‐rank sum test. We observed that there was a significant overlap between the results obtained by the limma and the Wilcoxon rank sum test, but the *t*‐test results were relatively deviated, which implied that the distribution of the expression data led to a result shift when using the *t*‐test (Supporting Information S1: Figure S1B). We further conducted the integrative analysis for differential methylation and expression by plotting the signed *t*‐statistics determined from differential analysis. As the integration is done at the level of statistics, there is no requirement for the DNAm and expression data to be matched (i.e., to come from the same individuals). The miRNAs were classified into four groups based on the intersection between the differential methylation and differential expression: hypermethylation‐upregulation (Hyper‐Up), hypermethylation‐downregulation (Hyper‐Down), hypomethylation‐upregulation (Hypo‐Up), and hypomethylation‐downregulation (Hypo‐Down) (Figure 1D). A number of studies had shown that promoter methylation led to the inactivation of tumor‐suppressor miRNAs, which was also observed in our analysis. The miR‐143‐3p, for instance, showed a strong negative correlation pattern between its expression and promoter methylation (*r* = −0.42 and *p* < 2.2e−16) (Figure 1E). This pattern has been confirmed by other studies. In addition, several other miRNAs that exhibited similar abnormal methylation and expression pattern in endometrial cancer had also been reported in the literatures, such as miR‐152, miR‐129‐2, and miR‐503, which indicated that our tool could capture the regulatory relationship of promoter methylation and its expression for miRNAs effectively.

Next, we further utilized the breast cancer (BRCA) cohort to demonstrate the effectiveness of this tool. This time we focused our analysis on the miRNA gene or precursor miRNA level. By using 825 normal and BRCA samples from TCGA, we constructed promoter methylation profiles for 1233 miRNA genes, with 810 of which were identified as differentially methylated with FDR < 0.05 (Supporting Information S1: Figure S2A). Further differential expression analysis for 1881 pre‐miRNAs in 1189 samples identified 668 differentially expressed miRNAs (Supporting Information S1: Figure S2B). The integrative analysis indicated many of these miRNA genes presented both differential methylation and expression (Supporting Information S1: Figure S2C). Several of these miRNA genes had been reported for their regulatory pattern between promoter methylation and expression, such as mir‐133a‐2, mir‐129‐2, and mir‐145, as well as mir‐195 and mir‐497. The scatter plot for expression and DNAm of mir‐145, mir‐195, and mir‐497 are presented in Supporting Information S1: Figure S2D–F, respectively. This global correlation pattern of their methylation and expression implies that miRNA promoters are frequent targets of aberrant DNAm in human cancers.

## 3 DISCUSSION AND CONCLUSION

Epigenetic regulatory mechanisms, including DNAm, play an important regulatory role in a variety of transcription products, including noncoding RNAs. Here, we developed a strategy to reannotate Illumina Infinium Methylation BeadChip probes from the early 450 K to the latest EPICv2 platforms at miRNA promoter loci and constructed miRNA methylation profiles, with the aim of determining the DNAm patterns for miRNAs in human diseases. The inaccuracy and incompleteness of the annotation of the promoter region have always hindered the in‐depth exploration of the transcriptional regulatory mechanism of miRNAs. To guarantee the performance of this tool, we evaluated several miRNA promoter annotation databases available till now, including the DIANA‐miRGen, mirTrans, miRStart, and FANTOM5; the FANTOM database was finally used as it offers the most detailed and reliable annotations on the miRNA promoter regions [7]. As more data on miRNA promoter annotation becomes available, we will update our tool accordingly.

We provide three different methods to construct methylation profiles for miRNAs: the method of taking the average of all CpG sites is currently the most widely used one. To avoid potential bias caused by the divergent methylation levels among different sites, we also provide the average of LOESS‐smoothing corrected methylation values. In addition, we also provide another method that uses the methylation level from the CpG site closest to the TSS as the representative as existing research indicates that the CpG sites in proximity to the TSS play a more significant role in gene expression regulation. Here, the range of the promoter region can be determined by users within 5000 bp up and downstream of the TSS, with 2000 bp up and downstream recommended. This tool also embeds different methods for screening the differentially methylated and differentially expressed miRNAs, and through integrative analysis, screens out miRNA genes that are highly related to their promoter methylation and expression, thus providing an effective approach for the efficient screening of disease‐related miRNAs.

Our tool will also be updated in alignment with future releases of the next generation of Illumina BeadChips. We also plan to extend this tool to DNAm datasets detected by other techniques, such as WGBS, RRBS, and MeDIP‐Seq. Currently, users may also convert these data into the Illumina platform format through tools, such as methyLiftover [8], thereby using this tool to construct the miRNA methylation profiles. In summary, miR2Meth provides a valuable toolkit written in R‐language for dissecting DNAm status at miRNA promoters by using the Illumina Infinium Methylation BeadChip data, and through integrative analysis with miRNA expression profiling, we can expect to get a better understanding of the biogenesis and transcriptional dysregulation of miRNAs in human cancers. miR2Meth is freely available on the GitHub website (github.com/zyangx/miR2Meth).

## 4 MATERIALS AND METHODS

The Illumina Infinium Methylation BeadChips have been the most widely used platform for genome‐wide DNA methylome profiling due to cost‐effectiveness and ease of use. We developed a strategy to reannotate the probes of Infinium BeadChips by mapping their genomic positions to miRNA promoter regions [9], which was validated by us recently in an application to TCGA 450 K DNAm data of lower grade glioma. Here, we extend our method to the next generation of EPIC and EPICv2 platforms. Briefly, the manifest files for the 450 K, EPIC, and EPICv2 platforms were obtained from the manufacturer’s website. The genomic coordinates of the CpG sites targeted by the array probes were converted to hg38 by using the liftOver tool from the UCSC genome browser. We next obtained the miRNA promoter annotation from the FANTOM5 database, which incorporates the detailed and reliable genomic coordinates of transcription start sites of 1357 miRNA genes based on the human genome assembly hg19, and then the coordinates were also converted to hg38. The BeadArray probes were assigned to miRNA promoters by calculating the overlap of their genomic positions using the GenomicRanges R package v1.56 [10]. The miRNA methylation profiles were constructed from the probes mapping into miRNA promoters with three different methods: averaging the *β*‐values of the probes mapping to each miRNA promoter; averaging the LOESS‐smooth fitted *β*‐values of the probes mapping to each miRNA promoter; or using the *β*‐value of the nearest CpG site to the TSS as representative. This package also provides functions to perform the differential methylation analysis of miRNAs by using the constructed profiles. In addition, by an integrative analysis of the differential methylation and differential expression for miRNAs, the impact of miRNA promoter DNAm on their expression can be determined.

## AUTHOR CONTRIBUTIONS

**Ying Shan**: Formal analysis; investigation. **Xiaoshan Rong**: Investigation; validation. **Andrew E. Teschendorff**: Conceptualization; writing—original draft, writing—review and editing. **Zhen Yang**: Conceptualization; funding acquisition; methodology; software; supervision; writing—original draft; writing—review and editing.

## CONFLICT OF INTEREST STATEMENT

The authors declare no conflicts of interest.

## ETHICS STATEMENT

This article does not contain any studies with human or animal materials performed by any of the authors.

## Supporting information

Supporting Information S1

## Data Availability

The study was based on the data available at the TCGA website (cancer.gov/tcga). The code used for this analysis is publicly available from the GitHub repository (github.com/zyangx/miR2Meth).

## References

[1] Bartel DP . MicroRNAs: genomics, biogenesis, mechanism, and function. Cell. 2004;116(2):281–297. 10.1016/s0092-8674(04)00045-5 14744438

[2] Gao Y , Feng C , Zhang Y , Song C , Chen J , Li Y , et al. TRmir: a comprehensive resource for human transcriptional regulatory information of miRNAs. Front Genet. 2022;13:808950. 10.3389/fgene.2022.808950 PMC8854293 35186035

[3] Baer C , Claus R , Plass C . Genome‐wide epigenetic regulation of miRNAs in cancer. Cancer Res. 2013;73(2):473–477. 10.1158/0008-5472.CAN-12-3731 23316035

[4] Peters TJ , Meyer B , Ryan L , Achinger‐Kawecka J , Song J , Campbell EM , et al. Characterisation and reproducibility of the HumanMethylationEPIC v2.0 BeadChip for DNA methylation profiling. BMC Genom. 2024;25(1):251. 10.1186/s12864-024-10027-5 PMC10916044 38448820

[5] Kozomara A , Birgaoanu M , Griffiths‐Jones S . miRBase: from microRNA sequences to function. Nucleic Acids Res. 2019;47(D1):D155–D162. 10.1093/nar/gky1141 PMC6323917 30423142

[6] Teschendorff AE , Marabita F , Lechner M , Bartlett T , Tegner J , Gomez‐Cabrero D , et al. A beta‐mixture quantile normalization method for correcting probe design bias in Illumina Infinium 450 k DNA methylation data. Bioinformatics. 2013;29(2):189–196. 10.1093/bioinformatics/bts680 PMC3546795 23175756

[7] de Rie D , Abugessaisa I , Alam T , Arner E , Arner P , Ashoor H , et al. An integrated expression atlas of miRNAs and their promoters in human and mouse. Nat Biotechnol. 2017;35(9):872–878. 10.1038/nbt.3947 PMC5767576 28829439

[8] Titus AJ , Houseman EA , Johnson KC , Christensen BC . methyLiftover: cross‐platform DNA methylation data integration. Bioinformatics. 2016;32(16):2517–2519. 10.1093/bioinformatics/btw180 PMC5006233 27153617

[9] Yang Z , Liu X , Xu H , Teschendorff AE , Xu L , Li J , et al. Integrative analysis of genomic and epigenomic regulation reveals miRNA mediated tumor heterogeneity and immune evasion in lower grade glioma. Commun Biol. 2024;7(1):824. 10.1038/s42003-024-06488-9 PMC11227553 38971948

[10] Lawrence M , Huber W , Pages H , Aboyoun P , Carlson M , Gentleman R , et al. Software for computing and annotating genomic ranges. PLoS Comput Biol. 2013;9(8):e1003118. 10.1371/journal.pcbi.1003118 PMC3738458 23950696

