# Supplementary material for: miR2Meth: An R package for dissecting DNA methylation heterogeneity at microRNA promoters
Source: Quant Biol. 2026 Oct 19;14(4):e70059. doi: 10.1002/qub2.70059 (PMC13681029; doi:10.1002/qub2.70059)
Supplement: Supplementary file 1 [file QUB2-14-e70059-s001.pdf]

Supplementary Information

miR2Meth: An R package for dissecting DNA methylation heterogeneity at microRNA promoters

Supplementary Figures

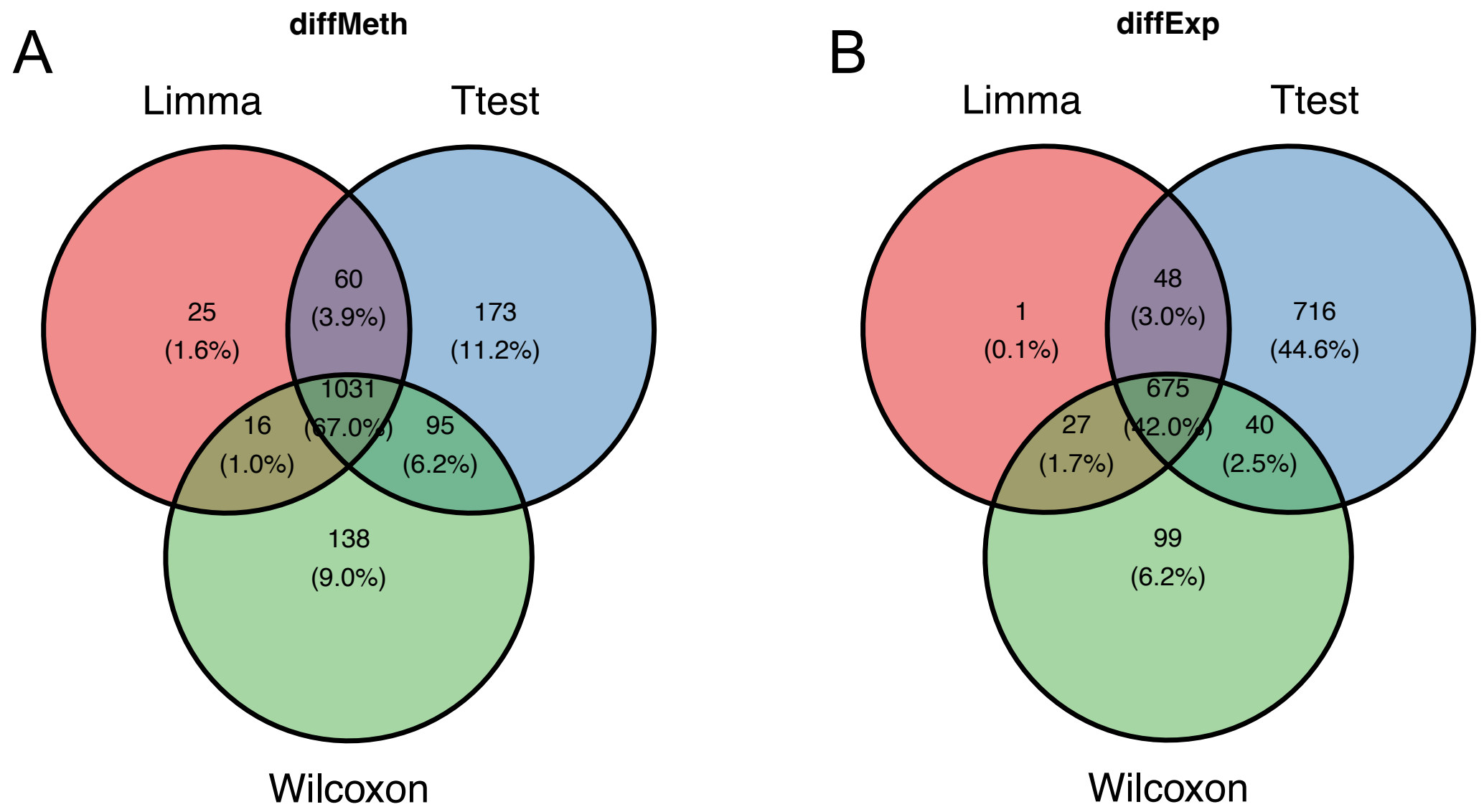

Supplementary Figure S1. Comparison for the results of differential methylation and differential expression analysis by using different tools.

(A) The Venn diagram shows the differences in the results of differential methylation analysis conducted by different methods. (B) The Venn diagram shows the differences in the results of differential expression analysis conducted by different methods.

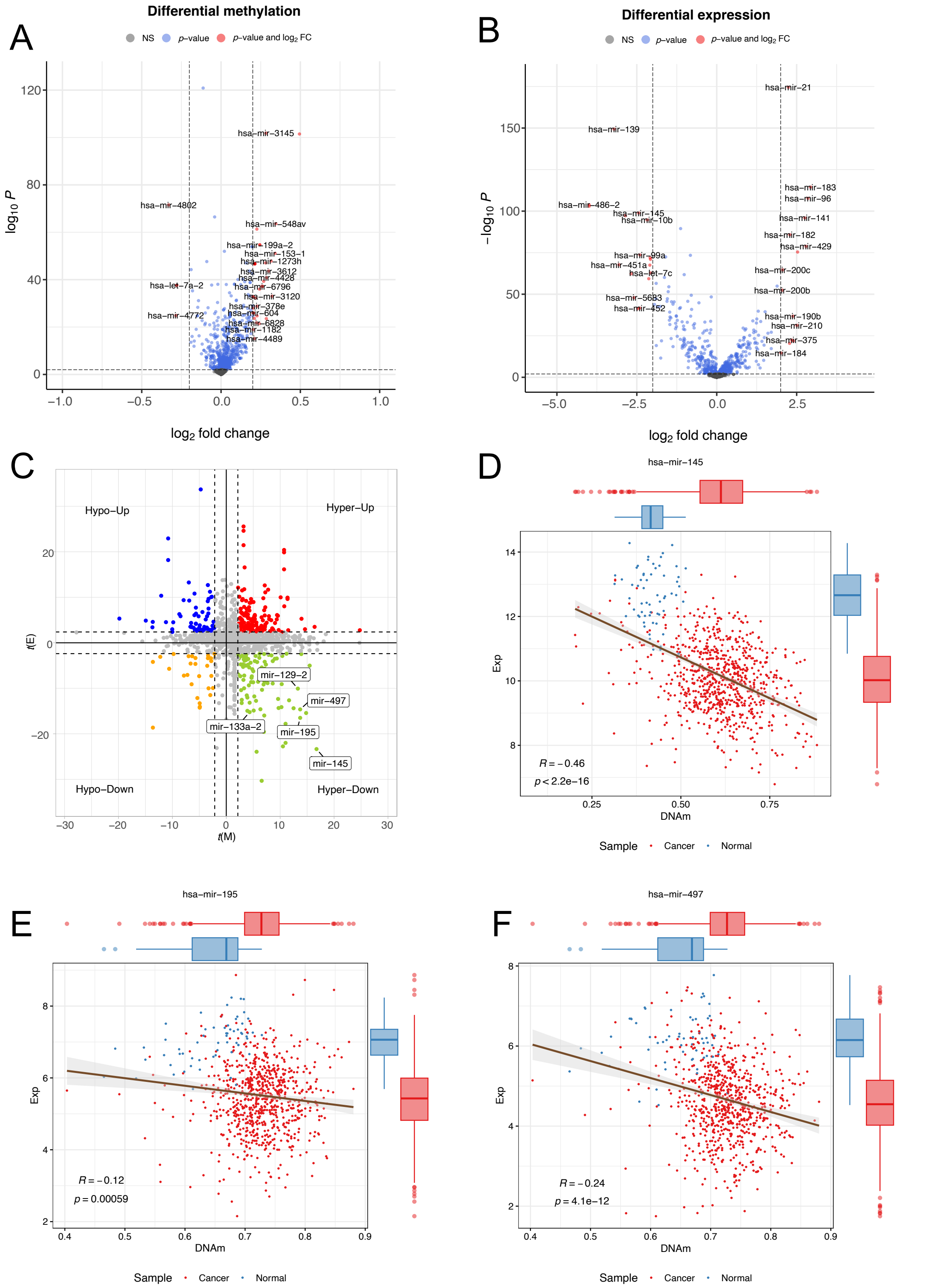

Supplementary Figure S2. Integrative analysis of differential promoter methylation and differential expression for miRNAs from the TCGA cohort dataset of breast cancer.

(A) Volcano plot for differential promoter methylation analysis. (B) Volcano plot for differential expression analysis. (C) Scatter plot of signed *t*-statistics generated from differential methylation analysis and differential expression analysis, four groups of

miRNAs that present significant differential promoter methylation and expression are labeled by different colors, some miRNAs that have been validated for their associations between promoter methylation and expression by previous studies are indicated. (D) Scatter plot for levels of expression and DNA methylation of has-mir-145. (E) Scatter plot for levels of expression and DNA methylation of has-mir-195. (F) Scatter plot for levels of expression and DNA methylation of has-mir-497. *p*-values are generated from Pearson correlation analysis.
